# Supplementary material for: Cold temperature induces a TRPM8-independent calcium release from the endoplasmic reticulum in human platelets
Source: bioRxiv. 2023 Jul 19:2023.07.19.549670. Preprint. [Version 1] doi: 10.1101/2023.07.19.549670 (PMC10370076; doi:10.1101/2023.07.19.549670)
Supplement: Supplement 1 [file NIHPP2023.07.19.549670v1-supplement-1.pdf]

bioRxiv preprint doi: https://doi.org/10.1101/2023.07.19.549670; this version posted July 19, 2023. The copyright holder for this preprint (which was not certified by peer review) is the author/funder, who has granted bioRxiv a license to display the preprint in perpetuity. It is made available under aCC-BY 4.0 International license.

Supporting information captions

Supplementary Figure 1. TRPM8 gene expression in megakaryocytic lineage during normal hematopoiesis. *Data were obtained from BloodSpot, a gene-centric database of mRNA expression of hematopoietic cells (Bagger et al., 2018). RNAseq was performed by Novershtern et al., 2011, source: GSE24759. The HSCs were identified as CD133-positive and CD34-dim (n=10), while Megakaryocytes as CD34+, CD41+, CD61+, and CD45-negative (n=7). Error bars indicate Mean ± SEM. Statistical analysis was performed using an unpaired Student t-test, where asterisks indicated a p = 0.009*

Supplementary Figure 2. RBC- and WBC-depleted platelet preparation shows CD45 and TRPM8 signals. *Agarose gel electrophoresis of PCR products from CD45-and CD235a (Glycophorin A)-depleted platelet preparation. PRP was depleted of CD45-positive cells using magnetic microbeads using AutoMACS sorter.* ***A****. PCR reaction using CD45 primers from CD45-positive cells, platelet preparations from two separate donors, and mock.* ***B****. PCR reaction using the TRPM8 1410F/1788R primer set. Arrows indicate the size of the expected amplicons: 300bp for the CD45 primer set; and 379 bp for the 1410F/1788R primer set.*

Supplementary Figure 3. TRPM8 receptor protein in human platelets by immunoblotting. A*. Western blot from HEK293T/17 cell lysates, transfected with GFP or TRPM8-GFP. Anti-TRPM8 (ACC-049) was used with or without blocking peptide (BLP-CC049). The expected size for TRPM8-GFP fusion protein is ~160kDa (two ways arrow). Blocking peptide subtracted line scan (BP sub line scan) was calculated by measuring pixel intensity along a line drawn down the lanes, normalizing to a background at high molecular weight, and subtracting the values measured for the corresponding lanes with blocking peptide (blue for TRPM8-GFP lane; orange for GFP). Arrow in the line scan indicates a full-length*

bioRxiv preprint doi: https://doi.org/10.1101/2023.07.19.549670; this version posted July 19, 2023. The copyright holder for this preprint (which was not certified by peer review) is the author/funder, who has granted bioRxiv a license to display the preprint in perpetuity. It is made available under aCC-BY 4.0 International license.

TRPM8-GFP protein. ***B****. Western blot of washed platelet lysates from three healthy donors. Line scan was calculated as in* ***A****. Arrows indicate potential TRPM8 protein.* ***C****. Representative images of random TRPM8-positive platelets population and CD45 (-/+) staining from one healthy donor by imaging flow cytometry. 20,000 events were measured for each sample. The scale bar is 7 μm.*

Supplementary Figure 4. TRPM8 agonists do not lead to activation of the human washed platelets after 1 hour of incubation. (A-B) *Human washed platelets were evaluated via flow cytometry.* ***A****.* Integrin αIIbβ3 activation in s*amples stained with PAC-1 fluorescent antibody (MFI normalized to vehicle).* **B.** Alpha granule release as seen from P-selectin externalization *(anti-P-selectin fluorescent antibody MFI normalized to vehicle). Initially, samples were pre-incubated with either vehicle DMSO or PF 05105679 (2 μM) for 5 minutes. Next, samples were treated with either vehicle (Ethanol), menthol (500 μM), WS-12 (2 μM) or icilin (100 μM) for 1 hour at either 22°C (white background) or 4°C temperature (green background). Values were normalized to those measured in platelets treated with the vehicle at 22°C. (****C-E****) Samples were evaluated via imaging flow cytometry.* ***C****. Percent microaggregates in samples treated the same as in A and B. (****D, E****) Percent spheroid (****D****) or discoid (****E****) cells in samples treated same as in A and B. Lines connecting data points indicate the same donor. Statistical analysis was performed using paired Student t-test, where asterisks indicated a p-value lower than 0.05 for *, and "ns" indicates s p-value >0.05. Symbols above brackets indicate paired comparison between treatment groups, and without bars indicate comparison to vehicle.*

Supplementary Figure 5. TRPM8 agonists do not lead to activation of the human washed platelets after 4 hours of incubation. (A-B) *Human washed platelets were evaluated via flow cytometry.* ***A****. Integrin αIIbβ3* activation in s*amples stained with PAC-1 fluorescent antibody (MFI normalized to vehicle).* **B.** Alpha granule release as seen from P-selectin externalization *(anti-P-selectin fluorescent antibody MFI normalized to vehicle). Initially, samples were pre-incubated with either vehicle DMSO or PF 05105679 (2 μM) for 5 minutes. Next, samples were treated with either vehicle (Ethanol), menthol (500 μM), WS-12 (2 μM) or icilin (100 μM) for 4 hours at either 22°C (white background) or 4°C temperature (green background). Values were normalized to those measured in platelets treated with vehicle. (****C-E****) Samples*

bioRxiv preprint doi: https://doi.org/10.1101/2023.07.19.549670; this version posted July 19, 2023. The copyright holder for this preprint (which was not certified by peer review) is the author/funder, who has granted bioRxiv a license to display the preprint in perpetuity. It is made available under aCC-BY 4.0 International license.

were evaluated via imaging flow cytometry. ***C.*** *Percent microaggregates in samples treated the same as in A and B. (****D, E****) Percent spheroid (****D****) or discoid (****E****) cells in samples treated the same as in A and B. Lines connecting data points indicate the same donor. Statistical analysis was performed using paired Student t-test, where asterisks indicated a p-value lower than 0.05 for *, and "ns" indicates s p-value >0.05. Symbols above brackets indicate paired comparison between treatment groups, and without bars indicate comparison to vehicle.* ***Supplementary Figure 6. (A-B)*** *Change in Calcium Green™-1 fluorescence levels baseline subtracted and normalized to maximum obtained after addition of calcium ionophore 7 µM A23187. HEK293T/17 cells transfected with TRPM8 (****A****) or empty vector (****B****), untransfected) were suspended in HEPES buffered saline containing either 0 mM* Ca^{2+} *and 100 µM EGTA (black) with vehicle DMSO, 2 mM* Ca^{2+} *with vehicle DMSO (gray) or 2 mM* Ca^{2+} *with 2 µM PF 05105679 (blue).* ***C.*** *Quantification of maximal calcium increase at 10˚C in HEK293T/17 cells, n=2.* ***D.*** *The overlay of the linear fit (dashed line,* R^2*=0.82) of the average negative control - the un-transfected HEK cells in 2 mM* Ca^{2+} *(same as in B, gray) and the average calcium response in washed platelets in 0 mM* Ca^{2+} *and 100 µM EGTA -containing Tyrode's buffer with vehicle DMSO (gray, same as Figure 7 C) or 5 µM thapsigargin (pink, same as Figure 8 C). Arrow indicates an apparent threshold for platelet activation at ~ 23˚C.*

bioRxiv preprint doi: https://doi.org/10.1101/2023.07.19.549670; this version posted July 19, 2023. The copyright holder for this preprint (which was not certified by peer review) is the author/funder, who has granted bioRxiv a license to display the preprint in perpetuity. It is made available under aCC-BY 4.0 International license.

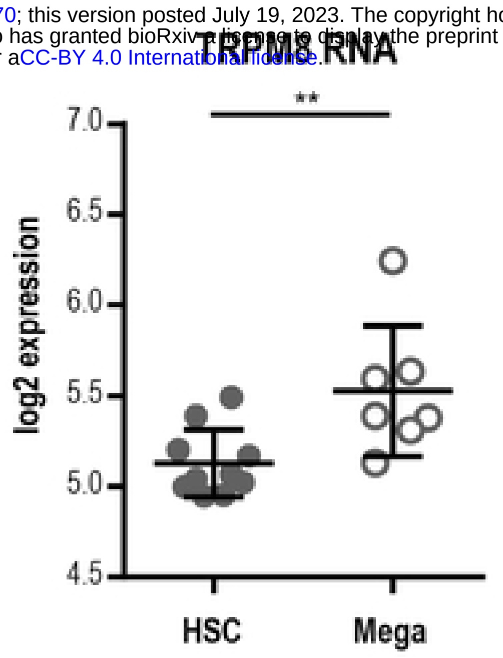

Suppl. Figure 1

bioRxiv preprint doi: https://doi.org/10.1101/2023.07.19.549670; this version posted July 19, 2023. The copyright holder for this preprint (which was not certified by peer review) is the author/funder, who has granted bioRxiv a license to display the preprint in perpetuity. It is made available under aCC-BY 4.0 International license.

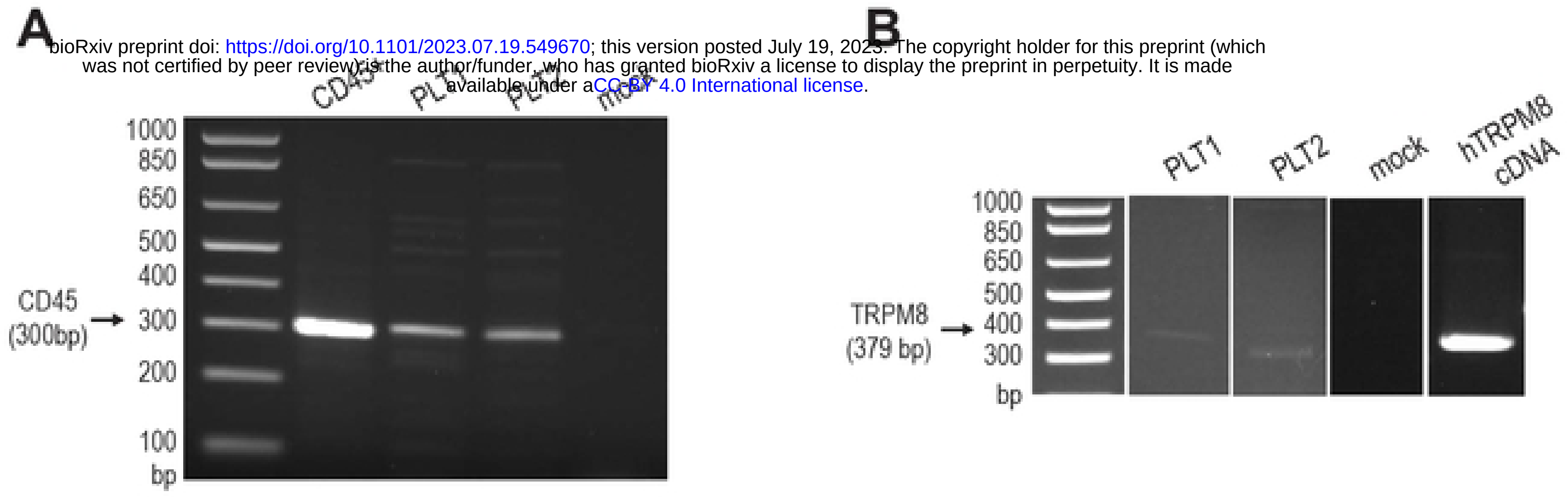

Suppl. Figure 2

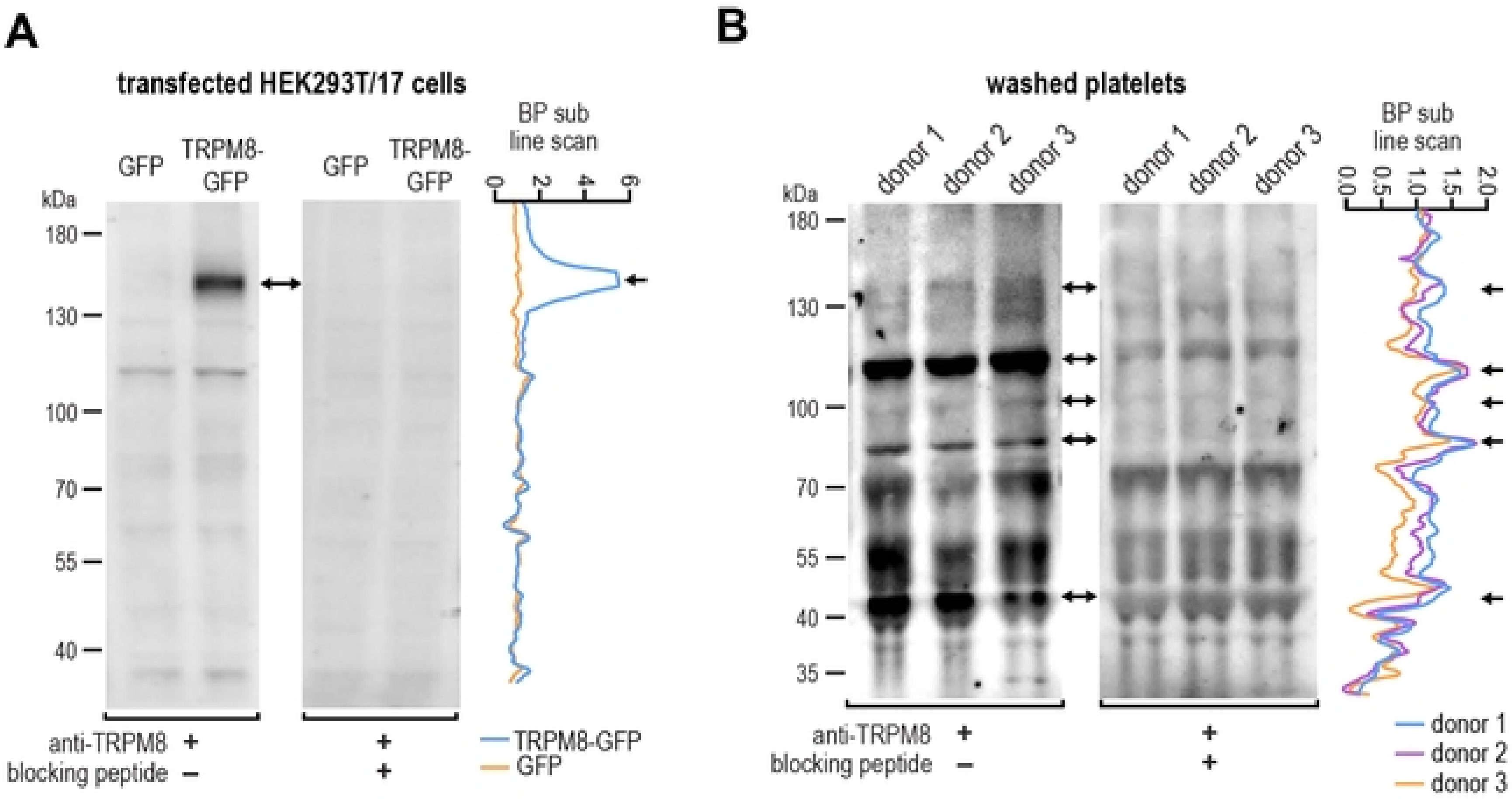

bioRxiv preprint doi: https://doi.org/10.1101/2023.07.19.549670; this version posted July 19, 2023. The copyright holder for this preprint (which was not certified by peer review) is the author/funder, who has granted bioRxiv a license to display the preprint in perpetuity. It is made available under aCC-BY 4.0 International license.

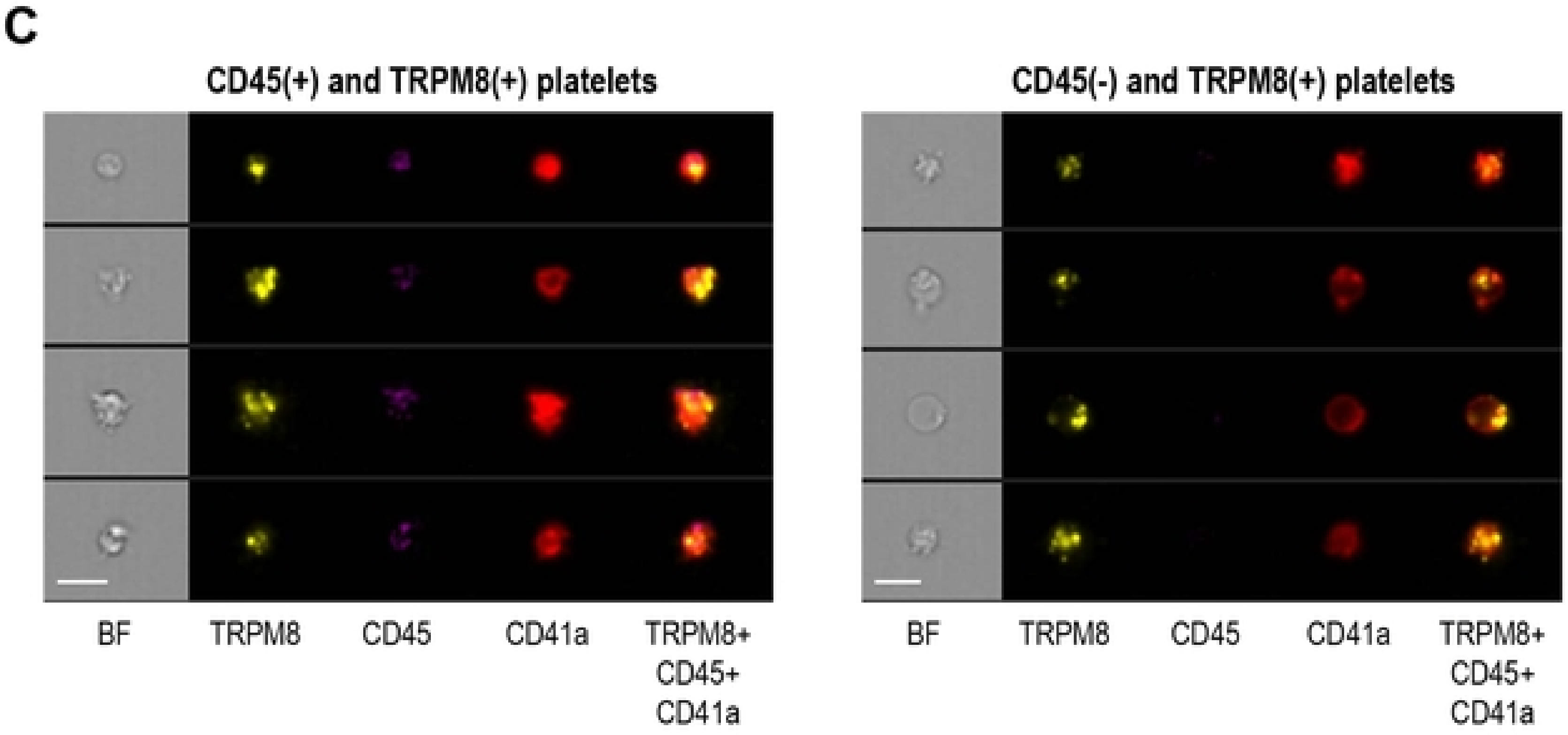

Suppl. Figure 3

1 HOUR INCUBATION

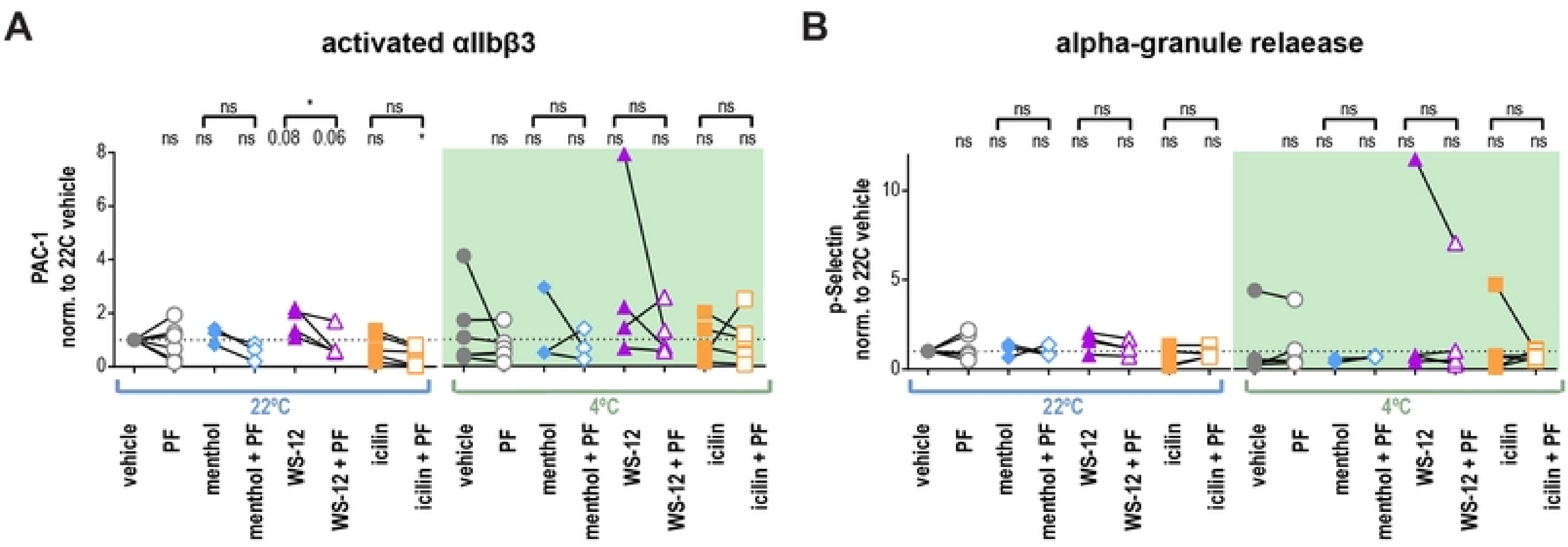

bioRxiv preprint doi: https://doi.org/10.1101/2023.07.19.549670; this version posted July 19, 2023. The copyright holder for this preprint (which was not certified by peer review) is the author/funder, who has granted bioRxiv a license to display the preprint in perpetuity. It is made available under aCC-BY 4.0 International license.

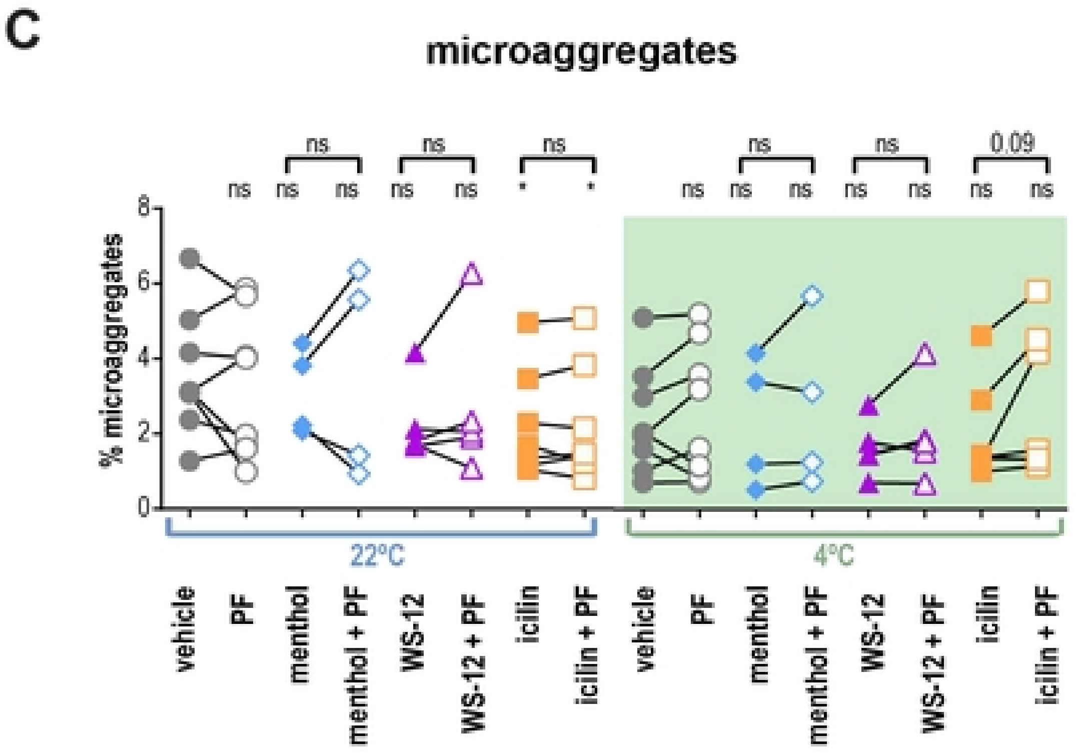

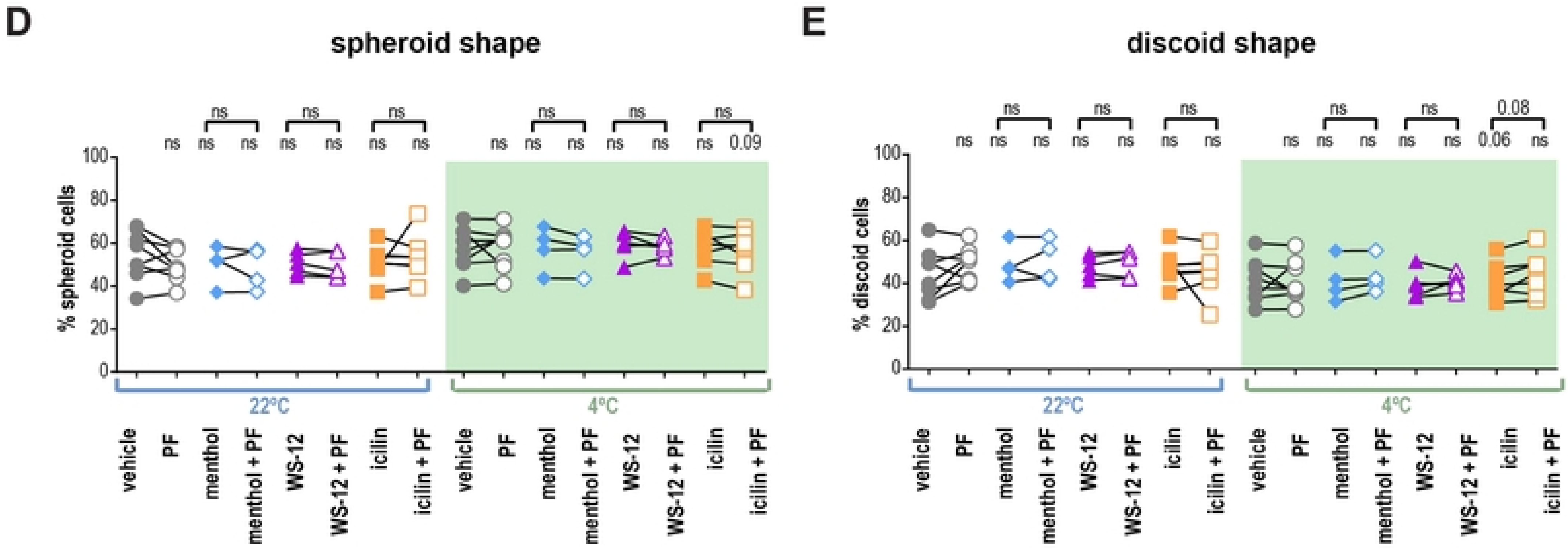

Suppl. Figure 4

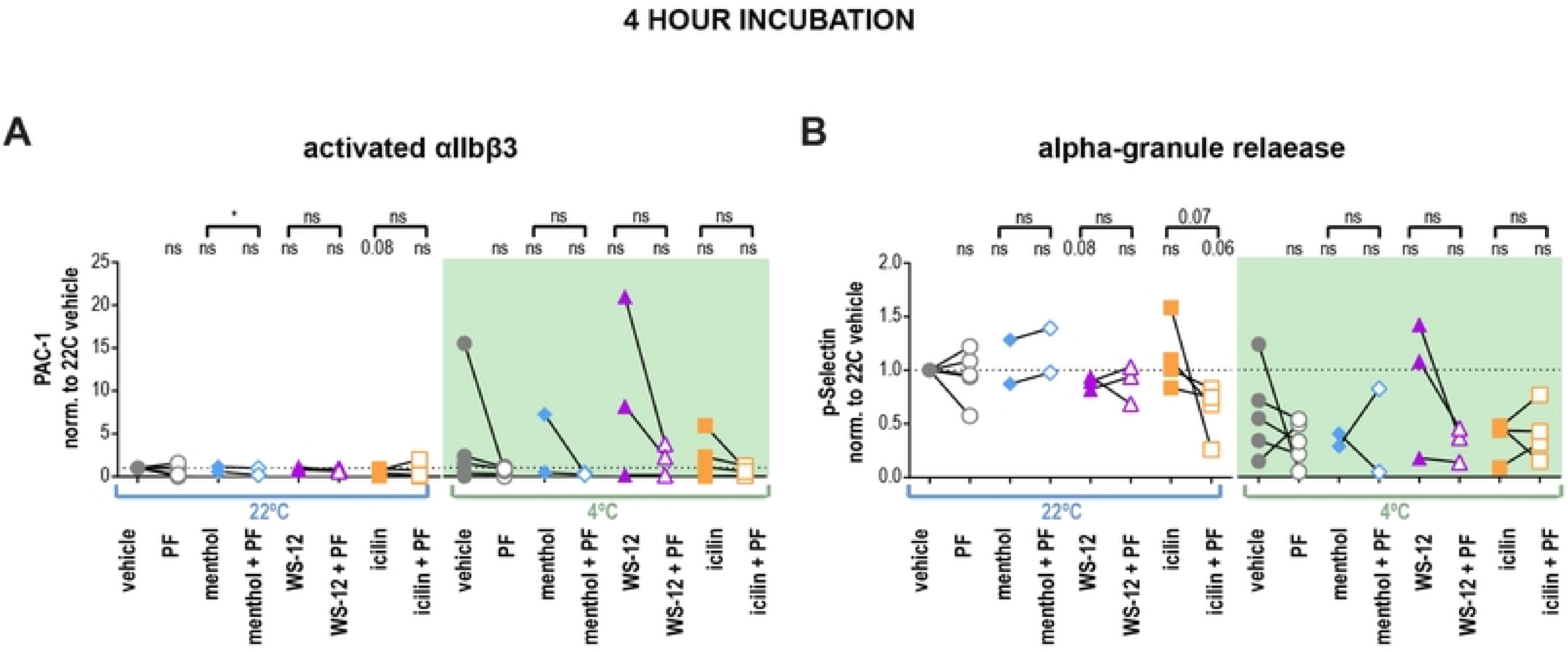

bioRxiv preprint doi: https://doi.org/10.1101/2023.07.19.549670; this version posted July 19, 2023. The copyright holder for this preprint (which was not certified by peer review) is the author/funder, who has granted bioRxiv a license to display the preprint in perpetuity. It is made available under aCC-BY 4.0 International license.

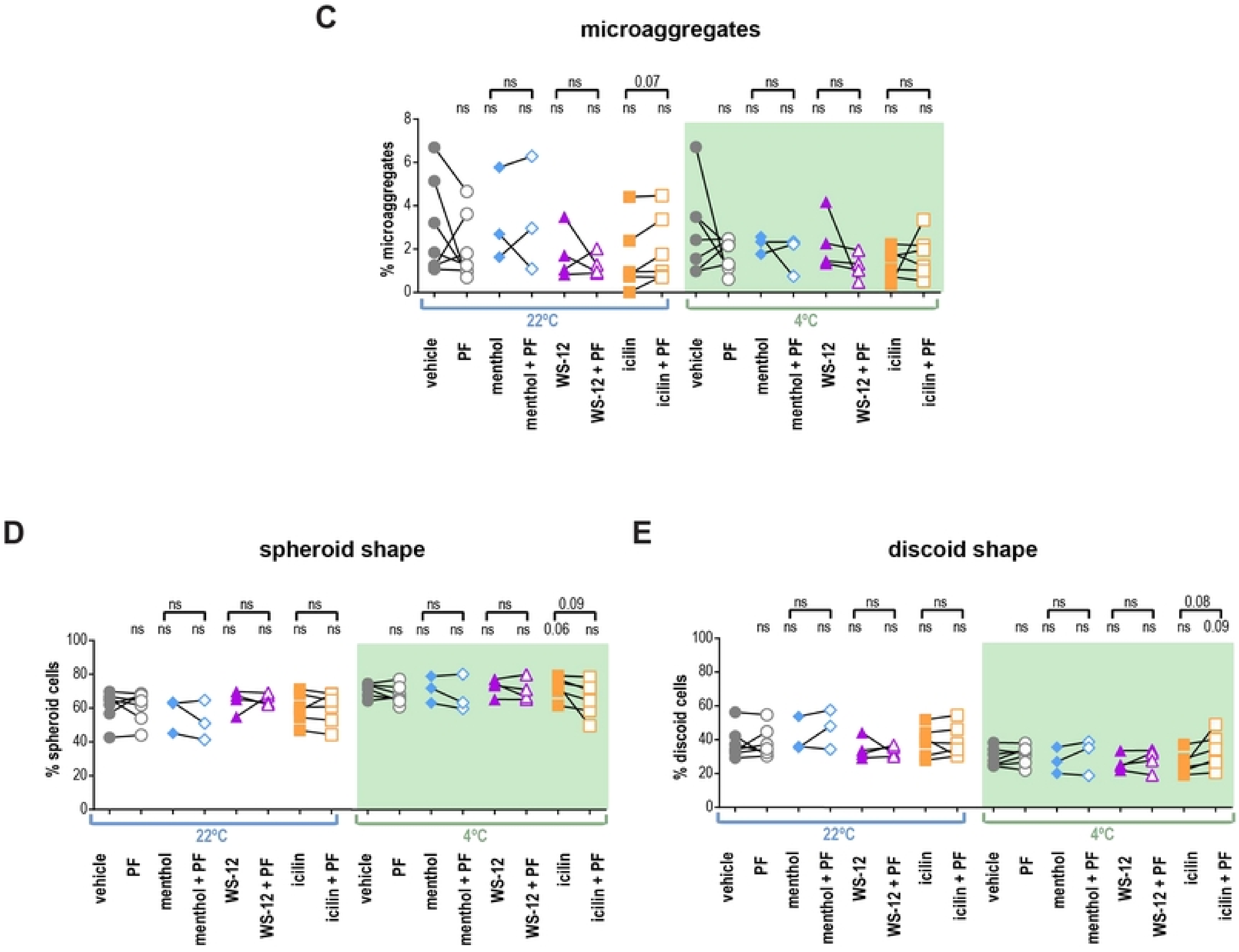

Suppl. Figure 5

bioRxiv preprint doi: https://doi.org/10.1101/2023.07.19.549670; this version posted July 19, 2023. The copyright holder for this preprint (which was not certified by peer review) is the author/funder, who has granted bioRxiv a license to display the preprint in perpetuity. It is made available under aCC-BY 4.0 International license.

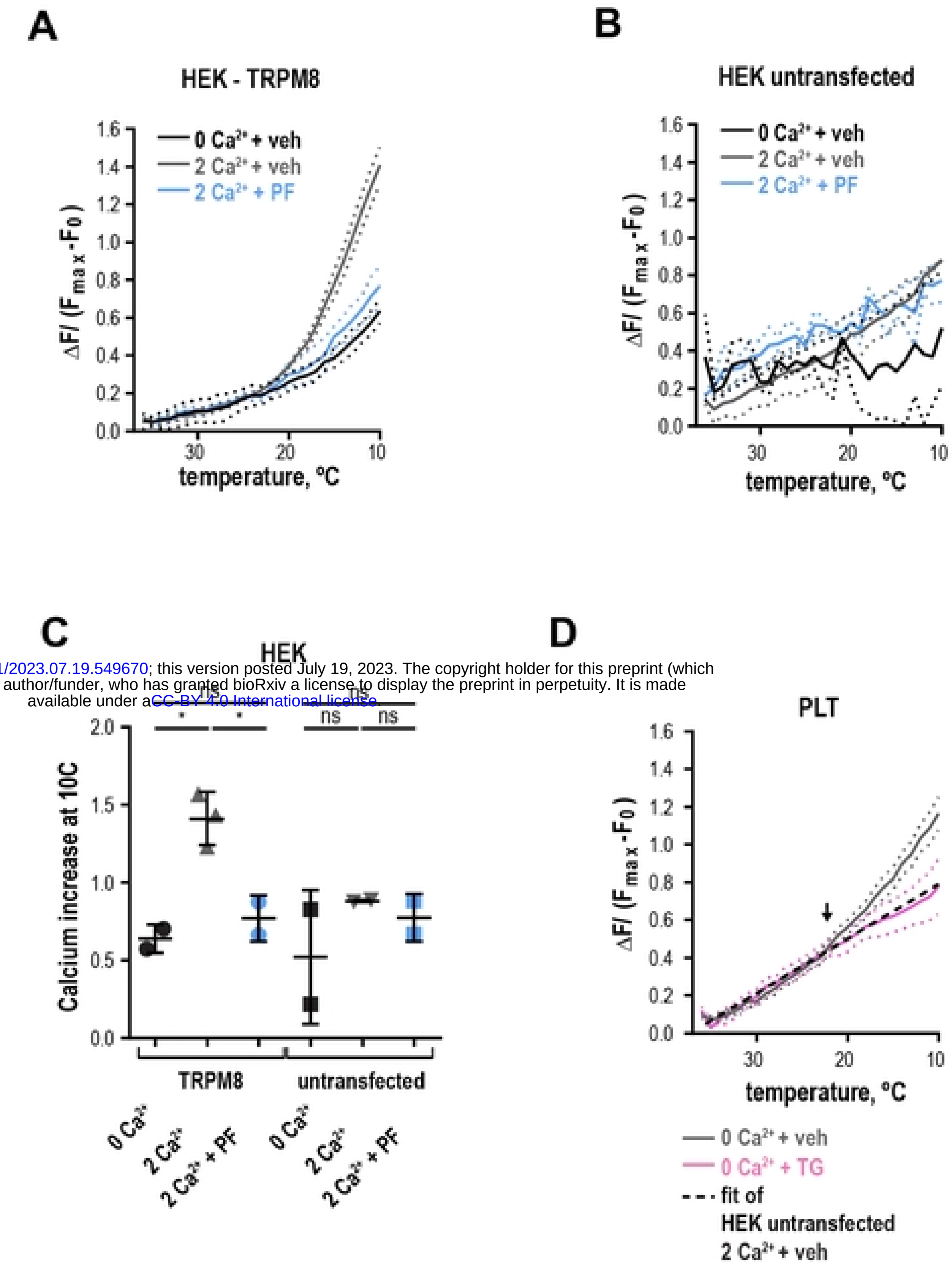

Suppl. Figure 6
